# Hidradenitis Suppurativa: The Influence of Gender, the Importance of Trigger Factors and the Implications for Patient Habits

**DOI:** 10.3390/biomedicines10112973

**Published:** 2022-11-18

**Authors:** Elia Rosi, Maria Thais Fastame, Gianmarco Silvi, Prisca Guerra, Giulia Nunziati, Antonella Di Cesare, Ilaria Scandagli, Federica Ricceri, Francesca Prignano

**Affiliations:** Department of Health Sciences, Section of Dermatology, University of Florence, 50125 Florence, Italy

**Keywords:** hidradenitis suppurativa, trigger factors, habits, gender, smoking, diet, sexuality, alcohol, clothing, pregnancy

## Abstract

Hidradenitis suppurativa (HS) is a debilitating, chronic, inflammatory skin disease primarily affecting apocrine gland-rich areas of the body. On the one hand, the presence of triggering factors—some identified, others only hypothesized—may initiate or perpetuate the pathogenic process of HS. In addition to cigarette smoking and diet, other trigger factors, including choice of clothing, are frequently observed in clinical practice. On the other hand, the presence of disease may influence habits of HS patients. Indeed, high incidences of sexual and sleep impairment have been reported in these patients. Consequently, alcohol and substance abuse may be a coping strategy for the emotional and psychological disease burden. Furthermore, a greater awareness of gender differences in HS may be important for dermatologists in their own clinical practice (i.e., pregnancy and breastfeeding). Consequently, in this loop interaction, comprehensive knowledge of all factors involved is crucial for the management of HS patients. Thus, the objective of this review is to (i) discuss the influence of gender on HS, (ii) summarize the most frequent triggering factors of HS and (iii) analyze the impact of HS on patient habits.

## 1. Introduction

Hidradenitis suppurativa (HS), also known as acne inversa or Verneuil’s disease, is a debilitating chronic skin disease of intertriginous areas [1].

“Taking care” of HS patients means not only knowing the pathogenetic mechanisms underlying the disease but also the habits of these patients, the triggering factors at the basis of this condition and the possible gender differences in the above aspects. Nowadays, disease treatment has to go beyond current standard therapy (either medical or surgical) and include the evaluation of patients’ unmet needs. Indeed, this approach may lead to (i) a greater knowledge of HS pathophysiology, (ii) a reduction in its exacerbations and (iii) an improvement in the quality of life of these patients.

Thus, in view of these considerations, the aim of this article is to provide the reader with an overview of the triggering factors of HS, the habits of HS patients and the gender differences in this condition. The innovation of this review is to summarize the knowledge on these issues and to highlight the bidirectionality of each factor in perpetuating or causing HS.

## 2. HS and Gender

Gender medicine has been a focus of research over the last decade. It evaluates whether gender is responsible for differences in disease epidemiology, pathophysiology, clinical manifestations and clinical and therapeutic approaches.

Epidemiological studies have found that the gender distribution of HS patients in different countries varies widely. Female predominance (with a female-to-male ratio of approximately 3:1) was observed in Europe and North America [2]. On the contrary, male predominance was noted in Asian populations (with a female-to-male ratio of approximately 1:2) [3,4,5,6,7,8]. The researchers hypothesized that genetics, hormones and differences in smoking habits (there was a significantly higher proportion of male to female current smokers in Eastern countries compared to Europe and North America) may play a role in the gender-distribution discrepancies [9,10,11,12].

The identification of gender differences in HS clinical characteristics has been the focus of numerous studies over the last few decades. Patient gender appeared to influence affected sites in HS. Indeed, female sex has been positively associated with breast and/or inguinal lesions (the front part of the body), whereas male sex was associated with greater gluteal, perianal and/or nape involvement (the back of the body) [13,14,15,16,17]. In addition, HS severity was positively associated with male sex according to Hurley stage [15,17], Sartorius score [16] and the International Hidradenitis Suppurativa Severity Score System (IHS4) [18]. Few studies have investigated gender differences in age of HS onset. According to some researchers, female HS patients had earlier disease onset than male patients [15,17,19,20]. Conversely, Morss and colleagues found no differences between males and females in terms of disease onset [21]. Furthermore, gender may influence the impact of HS on quality of life (QoL). In 24 HS patients, after 12 weeks of adalimumab, a better improvement in HIDRAdisk (a graphical tool able to describe HS burden) score was reported in males than in females [22].

Research interest in dermatologic disorders among sexual and gender minorities (SGMs) has grown rapidly in the last decades. Recently, Gomez et al., in a review article, provided the reader with the key evidence and recommendations for caring for SGMs with HS [23]. In this context, the management of HS in transgender individuals may be even more challenging, as masculinizing hormone therapy might worsen HS lesions [24,25]. 

Thus, in view of these considerations, knowledge of gender differences may provide practical management insights regarding HS patients.

## 3. HS and Race/Ethnicity

Relatively few studies have investigated racial/ethnic disparities in HS. Disease prevalence among African Americans was found to be higher than that among White populations [26,27,28,29]. Sachdeva and colleagues, in their systematic review, reported significant differences in race-specific prevalence of HS: prevalence rates were highest in African Americans and lowest in Hispanics/Latinos (intermediate among Caucasians) [30]. Obesity, a major health problem in African Americans, may play a role in developing HS; thus, it might represent a contributing factor to the increased prevalence of HS observed in the African American population [31]. HS patients with skin of color experience higher disease severity, lower QoL and a greater number of comorbid conditions [32]. Soliman et al. found that African American patients were more likely to have advanced disease and significantly lower socioeconomic status (SES) compared to non-African Americans [33]. In a retrospective 11-year cohort study in the US, the burden of hospital use was disproportionately associated with Black patients with HS [34]. Similar findings were confirmed by Kilgour et al., who noticed greater healthcare utilization and disease severity in HS patients of color compared to other groups [35]. Furthermore, racial differences may have an impact on HS management. Indeed, Robinson and colleagues found differences in treatment strategies between Black and White HS patients [36].

Interestingly, it was reported that the smaller variance in age at onset in Black HS patients might be explained by their significantly higher body mass indexes (BMIs—previously linked to increased HS severity and thus potentially to earlier age at onset of HS) [21]. Fairly recently, variation with race in serum cytokine levels was observed by McKay and colleagues. The researchers found that six cytokines (out of 25 analyzed) were lower in African American HS patients compared to other races [37].

Despite the above, racial and ethnic differences are minimally represented in HS randomized controlled trials. It is imperative that physicians and researchers promote racial/ethnic diversity during patient recruitment [38,39,40].

## 4. HS and Smoking

Tobacco smoking is one of the a major and well-known triggering factors in HS [41]. Many studies have demonstrated a significant association between HS and smoking. Konig et al. reported that the active cigarette smoker rate in HS patients was nearly 90% [42]. According to these findings, Breitkopf and colleagues found that the prevalences of smoking habits among men and women patients were 85% and 84%, respectively [43]. Dessinotti et al. showed that only 16% of HS patients had never smoked before [44]. Nevertheless, ex-smoking status does not seem to have a clear correlation with this disease [45]. Thus, in view of these considerations, active tobacco smoking might be considered a risk factor for HS. Indeed, this condition is associated with a two-fold increased risk of HS development [46]. Nevertheless, Saleem and colleagues underlined that, actually, the causal relationship between cigarette smoking and HS is unclear, because it would take 3300 smokers to have a new case of HS attributable to tobacco smoking [47]. On the other hand, cigarette smoking might also be a consequence of the disease, since patients may smoke to mitigate anxiety and depression, which are frequently associated with HS [48,49]. Cigarette smoking, as mentioned above, also might contribute to an explanation of the gender distribution of HS [9].

The role of tobacco smoking in relation to disease severity in HS patients has also been assessed. Previous investigations have suggested that (i) HS severity according to Hurley staging was not associated with smoking habits, whereas (ii) a correlation between smoking habits and the number of body areas affected by HS lesions existed [44,50]. Indeed, Sartorius at al. found that smokers had a higher disease severity (assessed by the modified Hidradenitis Suppurativa Score—HSS) than non-smokers, as this score takes into account the number of affected body areas [19]. Conversely, Chu and colleagues reported that Hurley score was significantly associated with smoking both in univariate and multivariate ordinal logistic regression analyses [51]. Cigarette smoking may also affect disease management, as non-smoking status was found to be linked to (i) increased odds of having a positive response to first-line therapy [52] and (ii) a greater rate of HS self-reported remission [53]. Thus, smoking cessation represents an essential step in HS treatment [54], even though the role of smoking on its etiopathogenesis is poorly understood.

**Figure 1:**
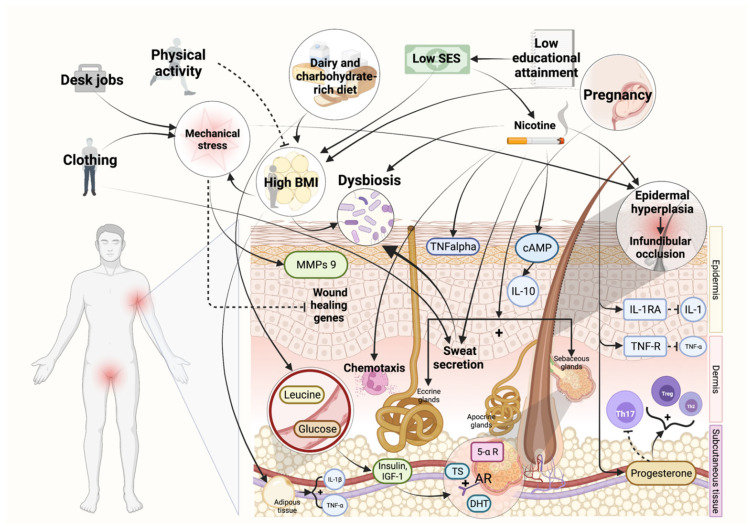
The main trigger factors involved in HS. For a detailed explanation, see the text. Image created with BioRender.com. Abbreviations: BMI, body mass index; SES, socioeconomic status; MMPs, matrix metalloproteinases; TNF—α, tumor necrosis factor alpha; TNF-R, tumor necrosis factor receptor; IL, interleukin; cAMP, cyclic adenosine monophosphate; IGF-1, insulin-like growth factor 1; AR, androgen receptor; TS, testosterone; DHT, dihydrotestosterone; 5-α R, 5-α-reductase; IL-1RA, interleukin 1 receptor antagonist. Information extracted from references [1,44,55,56,57,58,59,61,64,74,80,93,94,125,153,154].

Cigarettes contain many chemical substances, including nicotine, benzopyrene and arsenic dioxin-like compounds. Some of these elements may play a role in HS pathogenesis. Nicotine interacts with nicotinic acetylcholine receptors (nAChRs) of the pilosebaceous unit, thus inducing epidermal hyperplasia and, consequently, infundibular occlusion (Figure 1) [55]. Nicotine also induces neutrophil chemotaxis [56], keratinocyte release of TNF-alpha [44] and monocyte production of IL-10 (Figure 1) [57]. Furthermore, nicotine may modify cutaneous microbiome composition by promoting (i) Staphylococcus aureus growth [58] and (ii) Staphylococcus epidermidis biofilm formation (Figure 1) [59]. Arsenic and nicotine impair Notch signalling and skin homeostasis [60]. In addition, tobacco smoking modifies sweat gland activity: nicotine changes sweat composition and increases sweat secretion, leading to glandular duct plugging (Figure 1) [61]. Furthermore, benzopyrene increases the Th17 cell/Treg cell ratio by interacting with the aryl hydrocarbon pathway [62].

While there have been several studies on the association between HS and smoking, to date, the link between HS and vaping has not yet been investigated. It is plausible that vaping might influence HS course, as e-cigarette solutions often contain nicotine.

## 5. HS and Diet

Diet constitutes a wide field of interest and may play pathogenetic, preventive and therapeutic roles in some skin diseases, although its role in HS is not yet fully understood. In the literature, there is a growing body of evidence about the association between HS and inflammatory bowel disease (IBD) as well as HS and metabolic syndrome, all elements of which possibly suggest (i) an important role for diet, (ii) a common immunological pathway and (iii) gut microbiome alterations (dysregulation of the gut–skin axis) [63]. Furthermore, dietary habits may contribute to increased obesity, a preventable risk factor, which may trigger and exacerbate HS lesions (Figure 1) [64]. Obesity prevalence is higher among HS patients than among the general population [65], although the relationship remains unclear. Quite recently, a retrospective analysis of 1284 HS patients relative to age-, sex- and race-matched controls (enrolled between 1999 and 2019) was conducted by Wright et al. The researchers found that baseline BMI was higher among HS patients than controls (*p* < 0.001). This difference was larger for women than men [66]. Individual patient data from the PIONEER 1 and PIONEER 2 studies revealed that an increase in BMI (overweight and obese category) was associated with decreased odds of achieving Hidradenitis Suppurativa Clinical Response (HiSCR). Change in the IHS4 category and in abscess and nodule (AN) count was not significantly associated with BMI [67]. In view of the fact that obesity may have a negative impact on treatment efficacy for HS, obese paediatric HS patients might be at risk of recalcitrant disease [68]. In a cross-sectional analysis conducted in the USA which included 772 paediatric patients affected by HS, the prevalence of obesity among them was 68.7% (530/772) compared to 29.8% (392,367/1,315,332) for those without HS. After adjusting for sex, age and race, paediatric HS patients were 2.48 (*p* < 0.001) times more likely to be obese compared to those without HS [69]. A retrospective chart review estimated that only one third of HS overweight/obese paediatric patients were referred to adequate nutritional counselling, which is fundamental for the correct management of this chronic relapsing disease [70]. Sartorius and colleagues investigated the impact of BMI on disease severity by consecutively enrolling 251 patients (220 F, 31 M) over 7 years (January 2001–April 2008). On average, the patients were overweight (BMI = 28.3 ± 6.5). Most of the patients had Hurley II HS, and the study found a significant but weak positive correlation between BMI and the severity-index-modified Hidradenitis Suppurativa Score (HSS) (*p* = 0.036) [19].

**Figure 2:**
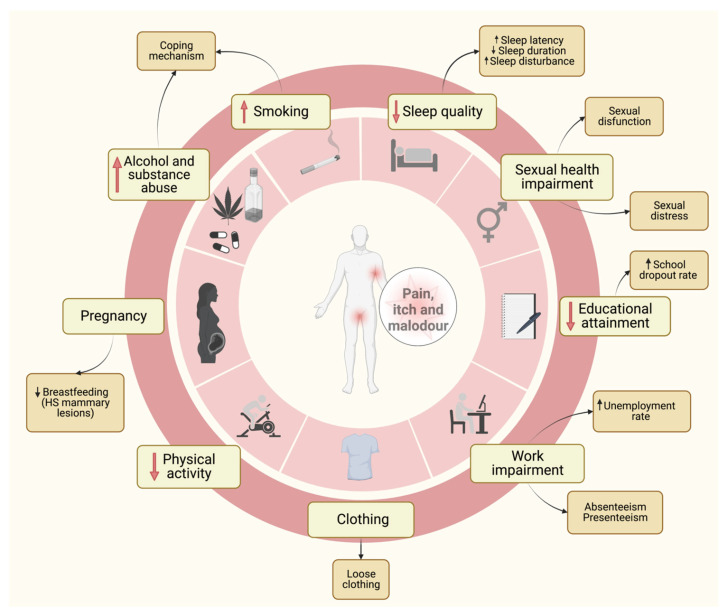
Main habits influenced by HS. For a detailed explanation, see the text. Image created with BioRender.com. Abbreviation: HS, hidradenitis suppurativa. Information extracted from references [72,86,87,112,131,132,133,134,135,136,137,152,157,158,168,169,173,174].

Thus, in view of these considerations, the objective of some papers has been to evaluate the possible impact of weight loss on HS course. Kromann and colleagues assessed the impact of body weight on the prevalence and severity of HS via a retrospective postal questionnaire distributed to 383 patients who had undergone bariatric surgery at their centre. Among 249 valid respondents, 45 patients had HS. Of these, 35 patients reported HS symptoms before surgery (and substantial weight loss), whereas, after surgery, 24 patients reported no/less severe HS symptoms, 7 reported unchanged severity and 4 reported increased severity [71]. Physical activity is critical to achieve and maintain an adequate body weight; nevertheless, pain and discomfort (due to HS lesions) present an obstacle for these patients (Figure 2) [72]. A retrospective descriptive study performed by Garcovich and colleagues showed that among HS patients undergoing bariatric surgery 11.4% of patients experienced worsening or de novo onset of HS lesions. This might represent an underestimated long-term complication of bariatric surgery [73]. Weight loss may be associated with symptom improvement in a substantial proportion of patients, but de novo onset of symptoms after weight loss suggests that this association may be multifactorial and involve numerous factors, including mechanical, microbiological and immunological ones [71]. Data from the literature showed that obesity is also to be considered a risk factor for malnutrition: adipocytes secrete chemokines that maintain a low-grade inflammatory state and promote oxidative stress by altering the transporters of some nutrients and causing depletion of antioxidants (Figure 1). Thus, malnutrition present at baseline in obese subjects might be an explanation for why bariatric surgery may not always lead to HS improvement [74].

A recent case–control study investigated anthropometric characteristics and adherence to the Mediterranean diet (MD) in 35 Sardinian HS patients (22 F, 13 M) relative to 35 healthy subjects matched for sex, age and geographic origin. Eight HS patients (22.8%) were classified as Hurley I, 16 patients (45.7%) as Hurley II and 11 patients (31.4%) as Hurley III. The prevalence of overweight and obese patients was significantly higher in the HS group than in the healthy controls (*p* = 0.02), even though Sardinia reports a lower prevalence of obesity than other Italian regions. The MD represents a healthy nutritional model (high intake of fruit, vegetables and fish, with a low intake of dairy and meat): HS patients showed lower adherence to the MD than controls [75]. A similar finding (regarding adherence to the MD) was reported in an observational study carried out on subjects of Neapolitan origin (41 patients with HS and 41 healthy controls). In addition, the researchers found a negative correlation between disease severity and PREvención con DIeta MEDiterránea (PREDIMED) score (a score that reflects adherence to the MD) [76]. According to these findings, a cross-sectional study involving 221 Spanish patients showed that greater adherence to the MD correlated negatively with IHS4 score. Furthermore, this study confirmed that vigorous physical activity was related to higher adherence to the MD, as observed in a healthy population [77]. In their cross-sectional survey, Dempsey et al. assessed the prevalence and the impact of dietary alteration on HS course. Of 242 surveyed, 183 (75.6%) respondents had made some dietary changes, in particular to their intake of gluten, dairy and refined sugars. Dairy consumption modification was common among patients in this study (43.8%) and significantly associated with HS symptom improvement (*p* = 0.05) [78]. An anonymous web-based questionnaire on diet and HS was distributed via social media to HS support groups and three North American clinics: 32.6% (n = 237/728) of participants identified HS-symptom-exacerbating foods (sweets, high-fat foods, bread/pasta, dairy) and only 12.0% (n = 89/744) identified alleviating foods, which included vegetables, fruit, chicken and fish [79]. Dairy (which includes casein, whey and natural androgens [65]) and high-glycaemic foods have a role in triggering the androgen-mediated follicular obstruction underlying the pathogenesis of HS (Figure 1). Considering this, intake reduction of these substances might reduce morbidity related to HS [80]. Cannistrà et al. performed a prospective study enrolling 12 HS patients (5 M, 7 F). Blood samples were collected from each patient to evaluate anti-*Saccharomyces cerevisiae* antigen (ASCA) IgG levels: all were found to have a specific immunological IgG reaction to ASCA (a predominant level of 22.9 ± 10.9 U/mL). Surgery followed by the subsequent elimination of foods containing yeast demonstrated a rapid stabilization of the disease and a slow regression of lesions [81]. Subsequently, 26 (70%) out of 37 patients who followed a yeast (*Saccharomyces cerevisiae*)-exclusion diet reported an improvement in HS symptomatology without any other treatment [82]. In a sub-analysis of a previous multicentre observational study, Damiani and colleagues investigated the impact of intermittent fasting during the month of Ramadan on HS patients: even though there was no significant change in the body weight of these patients, a statistically significant overall reduction in IHS4 was observed (*p* < 0.0001) [83]. Fairly recently, Sivanand et al., in their systematic review, examined the impact of patient-directed weight loss, dietary exclusion of brewer’s yeast, dairy restriction and bariatric surgery on HS severity; supplementation with vitamin D, riboflavin, turmeric and zinc gluconate was also investigated. All these interventions have been associated with improvement in HS symptoms [65,84].

Although larger multicentre randomized studies are needed to assess the actual potential impact of diet on HS, a growing body of evidence suggests its important role [85].

## 6. HS and Clothing

Although the choice of appropriate clothing (including underwear) has been scarcely considered in existing scientific papers, this aspect should be studied to improve the daily life of HS patients (reducing symptoms, such as itch and pain, and flare-ups). It is known that the combination of medical therapy and lifestyle modification represents the appropriate therapeutic approach for HS. Thus, although often not taken into consideration by either physicians or patients, the correct choice of clothing should be an integral part of HS management (Figure 2) [86]. In a recent survey, including a small number of HS patients, almost all of them reported that tight clothing and mechanical stress (related to pressure on skin) had worsened their HS lesions [87]. In addition, the characteristics and types of dressings used in HS patients [88] might influence choice of clothing.

HS is not considered an infectious disease. Nevertheless, the alteration of the skin microbiome may contribute to its pathogenesis, influencing its course: indeed, microbiome composition, especially in the intertriginous areas, contributes to malodour, local irritation and possible superinfections. In addition, dysbiosis and biofilm formation may also contribute to disease activity. Sweat plays a major role in worsening both HS lesions and symptoms (such as pain and itch). Furthermore, sweat may delay wound healing. For all these reasons, the use of absorbent devices, as well as the use of particular fabrics, may reduce the amount of sweat. Silver-containing fibers release nanosilver particles, thus contributing to the regulation of the local microbiome. Morand et al. reported the case of a 14-year-old patient suffering from acne vulgaris and HS (lesions were localized in the perineal region) previously treated with isotretinoin and subsequently with oral antibiotic therapy for 6 weeks without efficacy. The patient was advised to wear boxer briefs made of silver-coated textiles at night. He reported evident improvement in HS lesions. The use of silver-coated textiles (polyamide and elasthan), which has already emerged in recent years for its utility in the therapy of atopic dermatitis and epidermolysis bullosa, would seem to reduce skin colonization by *Staphylococcus Aureus* [89]. Thus, wearing clothing of specific materials should be considered in HS patient management with respect to antimicrobial, breathable and anti-irritant properties. Indeed, garments made from cellulose-derived rayon fibers or bamboo fibers may be useful for HS patients: they are soft but at the same time resistant materials, with excellent absorbent capacities, and they are able to maintain thermal homeostasis [86]. Nowadays, many patients feel the need to wear specific clothing. With the purpose of fulfilling their needs and helping them to manage their condition, it is possible to access “HidraWear”, an online platform where specific garments are offered for HS patients [90]. In addition, clothing may induce mechanical stress, which may contribute to the development of new lesions and/or the worsening of pre-existing lesions. Boer, in his case series of 14 obese HS patients, suggested the possibility of an HS-related Koebner phenomenon, for which new lesions may develop over time at the sites of clothing pressure/friction (mammary region, nape, inner thigh, waistband area) or at sites of skin-to-skin contact [91]. The authors suggested a two-hit model in which (i) obesity confers a basal susceptibility (ii) and a local susceptibility factor may promote ectopic lesion development [92]. The researchers hypothesized that the inflammatory substrate of HS, in which IL-17 plays a key role, may increase the predisposition to keratinocyte proliferation induced by mechanical stress (Figure 1) [93]. The latter may increase matrix metalloproteinase 9 (MMP-9) levels in keratinocytes and lead to the downregulation of several genes related to wound healing (laminin α5, connexin 43, interleukin α, keratinocyte growth factor, endothelin 1) (Figure 1) [94].

Thus, considering (i) the unmet needs of patients affected by a chronic relapsing disease, (ii) the role of mechanical stress and (iii) the properties of some types of fabrics on HS lesions, the choice of appropriate clothing should be considered in the management of this peculiar subset of patients.

## 7. HS and Sweating

Hyperhidrosis might influence HS course, since this condition predominantly involves apocrine-gland-bearing areas. To date, there is no evidence that hyperhidrosis plays a role in HS pathogenesis. Nevertheless, concomitant hyperidrosis may exacerbate HS symptoms, such as malodour and pruritus, which have negative impacts on patients’ quality of life [95,96]. Moreover, the reduction of sweating might positively influence microbiome composition and bacterial growth [97]. A recent systematic review [98] examined the efficacy and tolerability of hyperhidrosis treatments in HS patients. Botulinum toxin (BTX) A or B treatments proved to be effective in HS patients, whereas suction curettage and microwave-based energy devices showed no efficacy and/or safety in HS. BTX reduces eccrine and apocrine gland sweat production by inhibiting acetylcholine release from presynaptic vesicles. This mechanism may limit the tendency of follicular rupture, which is a contributing factor to HS pathogenesis [99]. BTX also exerts an inhibitory effect on neurogenic inflammation and nociceptive mast cell innervation, which is a possible explanation of the positive outcomes of BTX treatment in HS cases [97]. Interestingly, a prospective analysis conducted by Hua and colleagues reported a meaningful impact of BTX A treatment on the quality of life of patients with concurrent HS and hyperhidrosis (a condition significantly increased in subjects with HS) [100].

Antiperspirants and deodorants have been suggested as possible triggering factors for axillary HS lesions. Nevertheless, to date, there is scarce evidence for this. In 1980, Mustafa and colleagues reported that several patients affected by HS experienced a flare-up after the application of aluminium-containing deodorants [101]. Edlich et al. hypothesized that deodorants might trigger HS by reducing transepidermal water loss (TEWL) and consequently by promoting bacterial growth. Nevertheless, the researchers found that antiperspirants were not implicated in a significant change in TEWL of axillary skin [102]. Therefore, antiperspirants might facilitate HS lesion development through chemical irritation of axillary skin [103]. Interestingly, Morgan and colleagues [104] compared the prevalence of the use of deodorants between HS patients and a control group but did not find a statistically significant difference, and they concluded that these agents cannot be considered causative agents in HS pathogenesis.

## 8. HS and Shaving

Shaving is often believed to be a critical causal factor or contributing cause in the development of HS lesions. Thus, its avoidance is usually recommended in clinical practice. Nevertheless, there is little evidence to support this notion. Shaving was investigated as a triggering factor for HS as early as 1982, when Morgan and Leicester compared the practice of shaving the axillar and inguinal areas in 40 HS patients (prior to its onset) with 40 healthy controls. The authors found no significant difference between patients and controls and concluded that this habit was not primarily involved in disease initiation [104]. On the other hand, different studies have reported that shaving practice might be associated with either (i) progression of the disease [105] or (ii) its earlier onset [50,106]. It is not unusual and often observed in daily practice that patients themselves report shaving as an exacerbating factor. In a questionnaire-based survey of 110 HS patients, 13% of the subjects reported shaving of affected areas as an aggravating factor for the disease [107]. In a 2019 retrospective study on 40 HS patients, Kurzen et al. found that 57% of the subjects shaved or used to shave the affected areas, and three patients identified shaving as an aggravating factor. Several authors have hypothesized that both shaving and regrowing hair shafts, representing mechanical stress factors, might initiate the HS inflammatory cascade and promote follicular occlusion and rupture [94,105,108]. In accordance with this hypothesis, Meyer et al. reported the case of a patient in a vegetative state with classic HS in the genital area who developed histologically diagnosed HS lesions on his chin after daily, aggressive and traumatic shaving for years with an electric razor [109].

## 9. HS and Work

HS patients experience work impairment more often than subjects with other skin conditions. Indeed, HS is related to considerable physical and psychological disability, with a dramatic impact on daily activities, including those related to the occupational setting. Disease onset is usually between the second and third decade of life, which represents a very critical period for employment choices and opportunities [107,110,111]. As observed in a Danish cohort study of 100 HS patients, the unemployment rate within the working-age population (15–67 years of age) was more than six times higher than that reported for the general population in Denmark. In this study, 60.4% of the employed HS patients reported productivity impairment during work. The overall work impairment (absenteeism plus presenteeism) and the activity impairment, on average, were 26.6% and 32.7%, respectively. On the one hand, HS patients were more prone to absenteeism than control groups, with a mean percentage of absenteeism of 7%. On the other hand, the mean rate of presenteeism was 21.3% (Figure 2) [112]. Regarding HS impact on income, a retrospective cohort study in the USA reported significantly slower income growth in newly diagnosed patients with HS compared with controls and significantly lower annual income in HS patients than controls [113]. The main reason for work impairment in HS patients is functional disability, which determines incapacity to perform specific tasks. Symptoms, such as pain and pruritus, continuous flares and extensive scarring, represent major obstacles in carrying out daily life activities [114,115]. Another main problem in HS patients remains self-stigma regarding their disease. HS patients often feel embarrassed because of the multiple disfiguring and malodorous lesions, especially when located in visible sites of the body. They also find themselves impure and try to hide lesions with appropriate clothing. The most relevant consequence of self-perceived stigma is social isolation, with loss of working days and avoidance of interpersonal contacts [116]. On the other hand, work may also be considered a triggering factor in HS lesion development. To date, there is poor evidence concerning the possibility that different jobs may affect HS course. Nevertheless, it may be reasonably supposed that some jobs are associated with a major risk compared to others. For example, desk jobs may produce more static friction in some areas of the body, contributing to the triggering and maintenance of skin inflammation in predisposed subjects [94]. Moreover, HS patients display more difficulties in making a career and holding positions of responsibility due to their disease [117]. Thus, HS burden in the occupational setting has still to be extensively investigated. Nevertheless, it is certain that HS has crucial implications on overall working life.

## 10. HS and Educational Attainment

To date, there are still poor data on the influence of educational attainment on HS. If, on the one hand, HS might influence educational attainment, on the other hand, educational attainment might have an impact on diagnostic delay, comorbidities and therapeutic management of the disease.

Overall, it is well known that low-literacy patients report more chronic conditions than graduated patients [118,119]. Vazquez and colleagues, in their population-based study, reported that 18.3% of HS patients had less than 12 years of total schooling [120]. Bieniek et al. found that 40% of HS patients had an elementary education (including trade school) [121].

In our clinical experience, patients with disease onset at a younger age and with more severe conditions tend to drop out of school more often than the general population (data not published) (Figure 2). Indeed, functional disability, taboo and stigmatization, lack of self-esteem and social isolation [122] might have negative impacts on their individual academic careers.

## 11. HS and Socioeconomic Status

A multicentre cross-sectional reference study, evaluating SES in 1018 HS patients relative to 2039 sex- and age-matched dermatologic patients, found that Dutch patients affected by HS had a significantly lower SES compared with controls. Low SES, medium SES and high SES were observed in 46.4%, 39.0% and 14.6% of HS patients, respectively. In the multivariable analysis, low SES was associated with lower age, axillary involvement and high BMI in HS patients but not with HS severity or age of disease onset. The researchers even hypothesized that low SES (and its associated unhealthy lifestyle factors, such as smoking and obesity) might be an independent risk factor for HS [123]. More recently, the association between HS and lower SES (in comparison with matched controls) was confirmed in a cross-sectional survey-based study in the Northern Netherlands [124]. Low SES has a dramatic impact on healthcare and is significantly associated with poorer health and quality of life outcomes (Figure 1). In addition, physical and mental morbidity associated with HS may be an obstacle to maintaining employment, with consequent possible worsening of SES [125]. HS patients with low SES may encounter difficulties in attending outpatient appointments, including transportation issues, alternative childcare options and work flexibility. Patients often do not have the financial resources for medical fees, medical treatments and wound-care products [126,127]. Economic barriers also prevent HS patients from accessing healthcare structures and providers, thus leading to diagnostic delay [128].

For all these reasons, it is very difficult for clinicians to achieve therapeutic continuity as well as adequate clinical surveillance. In the management of HS patients, low SES represents a crucial challenge and requires not only solid medical knowledge but also an overall vision of patients’ unmet needs.

## 12. HS and Traumatic Life Events

According to a recent Belgian case–control study, negative life events were associated with increased risk of developing HS. In particular, the number of lifetime traumatic events, as well as childhood traumatic events (yes versus no) and the number of childhood traumatic events, were correlated with increased risk of developing HS. The association was strongest for emotional traumatic events as compared with physical traumatic events. Emotional traumas consisted of emotional neglect (being left alone or receiving insufficient affection) and emotional abuse (being belittled, teased, threatened verbally or unjustly punished) [129].

## 13. HS and Sexual Impairment

HS has been shown to have a significant detrimental effect on sexual health and intimate relationships. Indeed, pain, malodour, suppuration and the frequent involvement (also with active lesions or scarring) of erogenous areas in HS patients may contribute to this. Sampogna et al., analyzing the impact on sexual life through question 9 of the DLQI in 3485 dermatological patients, observed that HS was the skin condition associated with the greater sexual impairment [130]. Indeed, several studies reported (i) a high incidence of sexual impairment in HS patients [131,132,133], (ii) significantly greater sexual dysfunction (meaning impairment in the physical sphere) [134,135,136] and (iii) sexual distress (meaning impairment in the psychological sphere) [134,135,137] in patients affected by HS in comparison to control subjects (Figure 2). Interestingly, the evidence from available studies indicates that the disease impact on sexual functioning might vary depending on gender. According to some researchers, gender differences did not have an impact on sexual health in HS patients [136]. Conversely, some authors have observed higher levels of sexual distress in female HS patients compared with male patients (with similar disease severity) [133,134,137,138]. In a recent review and meta-analysis, Varney et al. found that female gender was associated with worse sexual impairment, whereas males were more affected by HS with respect to sexual functioning [139]. Furthermore, the authors suggested that the higher anogenital involvement (AGI) observed in male patients [120] might help explain the greater relative impact of HS on their sexual functioning [139]. In addition, some authors have found that patients with lesions on the lower abdomen had worse sexual functioning scores than patients without such lesions [134] and that active lesions in the groin and genitals were associated with sexual distress [137]. Nevertheless, in other studies, AGI did not correlate with sexual functioning measures (though it did with lower QoL) [132,135]. Other factors associated with greater sexual distress included subjective symptoms, such as pain [137,140] malodour [137] and suppuration [140], as well as later onset of the disease [132]. The association between impaired sexuality and disease severity is controversial. To date, the evidence does not support a correlation between disease severity and a higher risk of sexual distress or dysfunction [131,132,134,135,140]. Interestingly, while HS was not found to be associated with increased sexual impairment in previous research [132], in their cross-sectional study, Cuenca-Barrales et al. observed that the presence of a stable partner was a protective factor against sexual distress in women [137,140]. Furthermore, in another cross-sectional study, Cuenca-Barrales and Leyva observed that 94.3% of women and 80.8% of men reported that HS had a negative impact on their chances of having a relationship or finding a sexual partner, mainly due to fear of rejection [140]. Sisic et al. found a greater risk of intimate partner violence in patients with HS [141].

## 14. HS and Pregnancy

Physiological changes during pregnancy may have a variable impact on HS course [142]. A recent systematic review and meta-analysis (including 8 studies [53,143,144,145,146,147,148,149], 672 pregnancy cases and 164 postpartum cases) found that the patient-reported rates of HS improvement and HS disease worsening were 0.24 and 0.20, respectively. The percentage of patients reporting postpartum disease flare-up was 60% [150]. In their retrospective survey study, Prens and colleagues showed that 44.6%, 29.7% and 25.7% of patients experienced improvement (mostly in the first trimester), worsening (primarily in the third trimester) and no change in HS symptoms during pregnancy, respectively. Approximately 70% of women reported postpartum symptom deterioration, and complications (during and after pregnancy) occurred in 41% of patients [151]. Lyons et al. first investigated pregnancy and neonatal outcomes in 127 HS patients. The researchers found that rates of live births, likelihood of caesarean section and increased risk of gestational hypertension, preeclampsia, pregnancy complication or neonatal complication were not significantly associated with baseline HS severity. Interestingly, they observed (i) that in more than 10% of pregnancies women continued to use tobacco or marijuana during pregnancy, (ii) that the proportion of non-breastfeeding women was significantly higher in patients with HS breast lesions compared to those without HS breast lesions (Figure 2) and (iii) significantly higher average rates of gestational hypertension and pre-eclampsia compared to the US general population [152].

HS course may be influenced by pregnancy-related alterations in metabolic, immune and hormonal balances. Overall, the observed improvement in disease activity during pregnancy may be traced back to an increase in serum levels of (i) (in late pregnancy) interleukin (IL)-1 receptor antagonists (IL-1RA) and soluble tumour necrosis factor alpha (TNF-α) receptor (TNF-R), anti-inflammatory molecules neutralizing the effects of both IL-1 and TNF-α, respectively, and (ii) progesterone, which inhibits T helper (Th) 17 cell differentiation and promotes differentiation of Th2 and regulatory T (Treg) cells (Figure 1). On the other hand, pregnancy may worsen HS symptoms by increasing (i) the activity of sebaceous and eccrine glands and (ii) body weight, which in turn is associated with an increase in mechanical friction, insulin resistance and secretion (from adipocytes) of pro-inflammatory cytokines, including TNF-α (Figure 1) [1,153,154].

A quite recent survey study reported patient concerns regarding HS implications for fertility, pregnancy and childbirth, highlighting practice gaps in HS counselling [155].

## 15. HS and Sleep

Sleep impairment is an often-overlooked aspect that severely impacts the QoL of HS patients. Skin and sleep interact at multiple levels. Indeed, skin plays a role in both sleep onset and thermoregulation, whereas circadian rhythms (and their influence on cortisol levels) might affect skin symptoms [156]. Notably, both pain and itching, two prominent and disabling symptoms of HS, adversely affect sleep quality (Figure 2). In a cross-sectional study examining the prevalence and the characteristics of pruritus in HS patients, Vossen et al. observed that 70% of patients reported an impact of itch on different aspects of sleep [157]. Kaaz and colleagues, in their case–control study involving 108 HS patients and 50 controls, assessed the influence of pain and itch on sleep. The researchers found that, compared to controls, patients with HS did not have significantly more frequent insomnia (evaluated by the Athens Insomnia Scale—AIS) but did have significantly more sleep disturbances (evaluated by the Pittsburgh Sleep Quality Index—PSQI). Specifically, pain played a crucial role in determining subjective sleep quality, sleep duration and daytime dysfunction (Figure 2) [158]. It has also been suggested that HS might be related to a higher risk of obstructive sleep apnoea (OSA), a clinical condition characterized by airway collapse and subsequent hypoxia during sleep. In this regard, both these conditions share common risk factors, such as obesity and smoking. Furthermore, OSA might further worsen already poor sleep quality in HS patients. In a cross-sectional study analyzing the prevalence of OSA and non-OSA sleep disorders (nSD) in a large Israelian HS cohort, a significant association between HS and both OSA and nSD groups was found. This association persisted, although weakened, after multivariate analysis of risk factors [159]. According to these findings, other studies observed higher odds of OSA incidence in HS patients, thus confirming a link between these two conditions [160,161].

## 16. HS and Alcohol and Substance Abuse

The relation between HS and alcohol, cannabis and other drug use is still under investigation. Different studies found that alcohol consumption did not impact HS course [162,163,164]. Fairly recently, a study on a population of 48 post-surgery Hurley III HS patients observed a slight but statistically significant risk of local recurrences related to alcohol consumption. The authors concluded that alcohol abstinence might help reduce disease relapses [165]. Subsequently, the effect of alcohol avoidance was investigated by Dempsey et al. in a cross-sectional study. The authors analyzed the dietary alterations of HS patients. They observed a reduction in alcohol consumption in 37.1% of the participants. Nevertheless, this modification was not significantly associated with disease improvement [78]. Theut Riis et al., considering a cohort of blood donors, found that HS donors drank less wine but more spirits (less expensive than wine) than blood donors without HS. They suggested that this observation might be indicative of HS patients’ lower SES. No difference in beer consumption was reported [166]. Therefore, to date there is scarce evidence regarding the impact of alcohol consumption on HS. On the other hand, several findings have suggested that HS might instead influence alcohol and other substance misuse. Many studies have reported a higher prevalence of (i) alcohol [167,168,169,170,171] and (ii) substance abuse in HS patients [167,168,169,172,173]. According to Garg et al.’s findings, the prevalence of substance-related disorders among patients with HS was 4.0% and 6.5% for alcohol and drugs, respectively. Specifically, the most common forms of substance misuse were alcohol (47.9%), opioids (32.7%) and cannabis (29.7%) [168]. Some authors have hypothesized that HS patients might use the aforementioned substances for their analgesic effects and to cope with the emotional and psychological disease burden (Figure 2) [131,168,169,173,174]. In a multicentre French study, Lesort et al. found a significantly greater prevalence of cannabis use in HS patients (37%) compared to the general population (11%) and psoriasis patients (11.6%). Interestingly, HS patients denied using cannabis for pain relief. Nevertheless, a significant inverse association between pain scores and cannabis use was observed. In addition, the researchers, proceeding from the observation that most patients (70%) started using cannabis prior to disease onset, hypothesized a possible role of this substance as a trigger factor [173]. In a US retrospective study, Puza et al. observed that 60% of HS patients received opioids for pain management [175]. Aldana et al. hypothesized that increased risk of opioid abuse may be consequential for the prescription of substances with a high potential for abuse [174].

## 17. HS, Tattooing and Body Art

A scarcely investigated sphere of HS patients’ life is the prevalence of tattooing, piercing and other body-art practices. Quite recently, a French questionnaire-based study observed that subjects with HS were more likely to have tattoos or body piercings (BPs), regardless of gender or age, compared with either the general French population or patients with other skin conditions. No complications were reported in relation to their tattoo(s) or BPs in the study population [176]. The authors further hypothesized that tattoos and BPs might be a way for patients to hide or embellish scars and reclaim control of their bodies. Furthermore, they may have a positive impact on the patient’s psychology and body-image perception. Nevertheless, Kjærsgaard Andersen and Alsing, in their reply, pointed out that this observed association did not consider confounding factors, such as the higher prevalence of tattoos in younger populations with lower educational levels and SES (known epidemiological features of HS patients) [177].

## 18. Conclusions

Gender medicine plays an important role in the process of providing personalized care in HS. Knowing the triggering factors allows dermatologists to educate their HS patients about avoiding them to prevent flare-ups. Being aware of the habits of HS patients makes it easier to manage their disease. Furthermore, every physician should be aware of the bidirectionality of each factor in perpetuating or causing HS.

## Data Availability

Not applicable.
